# Expression of multidrug resistance proteins in invasive ductal carcinoma of the breast

**DOI:** 10.3892/ol.2014.2435

**Published:** 2014-08-11

**Authors:** WEIQUAN LI, MAOMIN SONG

**Affiliations:** Department of General Surgery, Beijing Tiantan Hospital, Capital University of Medical Sciences, Beijing 100050, P.R. China

**Keywords:** breast neoplasm, multidrug resistance-related protein, topoisomerase2α, thymidylate synthase, glutathione-s-transferase π, multidrug resistance, immunohistochemistry, p-glycoprotein

## Abstract

Chemotherapy is commonly used for the treatment of breast cancer. However, the resistance to chemotherapeutic agents, often mediated by multidrug resistance (MDR) mechanisms, is a common occurrence. The present study examined the expression of several MDR-related proteins (MRPs) in invasive ductal carcinoma (IDC) of the breast, and assessed their association with clinicopathological variables and their prognostic significance. In addition, immunohistochemistry was used to measure the expression of MRP, p-glycoprotein (P-gp), topoisomerase 2α (Topo2α), thymidylate synthase (TS) and glutathione-*S*-transferase π (GST-π) in 156 resected IDCs of the breast. Pearson’s χ^2^ test and Spearman’s correlation coefficient were used to analyze the association between MDR protein expression and several clinicopathological variables. The association between each of the five MDR proteins was also examined. Furthermore, Kaplan-Meier analysis and Cox regression modeling were used to assess overall survival. The expression of MRP, P-gp, Topo2α, TS and GST-π was detected in 20.5% (32/156), 25.0% (39/156), 84.0% (131/156), 41.7% (65/156) and 41.0% (64/156) of cases examined, respectively. No correlation was identified between MRP and Topo-2α and the clinicopathological variables examined. By contrast, P-gp (χ^2^=20.226; P<0.0001) and GST-π (χ^2^=35.032; P<0.0001) were found to positively correlate with tumor grade. In addition, staining for TS was associated with axillary lymph node metastasis (χ^2^=42.281; P<0.0001). The expression levels of P-gp and GST-π were found to be significantly correlated (r= 0.319; P<0.0001). Furthermore, GST-π expression was elevated in estrogen receptor-negative breast cancer (χ^2^=17.407; P<0.0001). Tumor histological grade, in addition to TS and GST-π expression, were significant predictors of a poor survival outcome. TS and GST-π are consequently useful prognostic biomarkers in IDC, therefore, when establishing a personalized chemotherapeutic plan, the expression of MDR proteins must be considered.

## Introduction

Invasive ductal carcinoma (IDC) of the breast is a malignant disease, which affects numerous females worldwide (1). Chemotherapy in the neoadjuvant and adjuvant settings is widely administered for the treatment of breast cancer (2). However, despite its success, resistance to chemotherapeutic agents is a common occurrence that is often attributable to mechanisms of multidrug resistance (MDR) (3,4). Although proteins that mediate this resistance mechanism have been identified and have the potential to serve as biomarkers or prognostic indicators of outcome, the function of these MDR-related proteins (MRPs) in IDC of the breast has not been extensively investigated.

The critical proteins that mediate MDR in tumors include MRP, p-glycoprotein (P-gp), topoisomerase 2α (Topo2α), thymidylate synthase (TS) and glutathione-*S*-transferase π (GST-π). A number of these proteins have been investigated in other tumor types, including esophageal, colorectal and endometrial cancer. In numerous instances, resistance is achieved by an increased efflux of chemotherapeutic agents out of the tumor. Occasionally, this is an acquired problem, as while certain tumors are initially responsive, they become resistant following prolonged treatment. In other cases, tumors fail to respond to therapy at all, a mechanism known as *de novo* resistance (5).

The function of these MRPs in IDC of the breast has not been extensively investigated. Notably, the expression of MRP, P-gp, Topo2α, TS and GST-π exhibit the potential to serve as biomarkers for the disease and have prognostic significance. The aim of this study was to examine the expression of MRP, P-gp, Topo2α, TS and GST-π in breast IDC, and assess their association with clinicopathological variables, as well as their prognostic significance. The results may aid clinicians in the design of unique treatment regimens for each individual patient.

## Materials and methods

### Patients and specimens

Samples were obtained from patients with IDC of the breast who underwent primary surgery at Beijing Tiantan Hospital, Capital University of Medical Sciences (Beijing, China) between 2005 and 2007.

Prior to patient enrolment, the expression of MRP, P-gp, Topo2α, TS, GST-π, ER, PR, HER2 and pP53 was analyzed by staining the excised tumor tissue. In total, samples from 156 female patients were analyzed. The patient age ranged between 32 and 75 years (median age, 52 years). No distant metastases were detected in any patients during pre-operative examination. Lumpectomy and axillary dissection was performed in 20 cases, radical mastectomy in 24 cases and modified radical mastectomy in 112 cases. Immunohistochemical staining was performed at Beijing Tiantan Hospital. The clinicopathological data are shown in Table I.

The follow-up period consisted of the time from the first day following surgery until December 2012. Survival time was calculated from the first day following surgery until mortality or the last follow-up. The study was approved by the ethics committee of Beijing Tiantan Hospital Affiliated to Capital Medical University (Beijing, China). All patients provided written informed consent.

### Immunohistological analysis

All tumor tissues were fixed in neutral buffered 4% formaldehyde and embedded in paraffin. Immunohistochemical staining was performed using an avidin-biotin peroxidase system (SP-9000 kit; Zhongshan Goldenbridge Biotechnology Co., Ltd., Beijing, China). All primary antibodies and reagents were purchased from Beijing Zhongshan Goldenbridge Biotechnology Co., Ltd. The following monoclonal antibodies were used: MRP (OCRL-1), P-gp (C494), Topo2α (3F6), TS (TS106), GST-π (LW29), ER (1D5), PR (1A6), HER2 (CB11) and p53 (DO7). Antigen retrieval for all proteins, with the exception of GST-π, was performed in citrate buffer (pH 6.0) by autoclaving for 180 sec at 100°C. Staining was performed using the LabVision Autostainer360 System (Maixin. Bio. Co. Ltd., Fuzhou, China).

Positive staining of the tumor cells was determined by the appearance of a brown-yellow color. Protein staining scores were defined as follows: 0, negative or <10% of tumor cells stained positive; +1, 10–25% of cells stained positive; +2, 26–75% of cells stained positive; and +3, >75% of cells stained positive. HER-2 overexpression was scored based on the degree of membrane staining according to the manufacturer’s instructions for the HercepTest (6). The following parameters were applied for the assessment of HER-2 expression: 0, no membrane staining or membrane staining in <10% of tumor cells; 1+, faint/barely perceptible partial membrane staining in >10% of tumor cells; 2+, weak to moderate staining of the entire membrane in >10% of tumor cells; and 3+, marked staining of the entire membrane in >10% of tumor cells. A score of either 0 or 1+ was considered negative and scores of 2+ and 3+ were considered positive for HER2 overexpression. For each sample, ≥10 fields (magnification, ×200) were randomly selected for analysis, whereby >500 positive cells were counted, and the average was calculated. Scores were assigned independently by two different pathologists. In the case of a discordant result, additional fields were counted and analyzed.

### Statistical analysis

Statistical analyses were carried out using SPSS 19.0 software (SPSS Inc., Chicago, IL, USA). Pearson’s χ^2^ test and Spearman’s correlation coefficient were used to analyze the association between MDR protein expression and clinicopathological variables, as well as ER, PR, HER2 and p53 status. Similar calculations were performed to assess the association between the five MDR proteins analyzed in the study. Survival analysis was used to determine prognostic significance using Kaplan-Meier analysis and the Cox regression model. P<0.05 was considered to indicate a statistically significant difference.

## Results

### Patient characteristics

A total of 23 (14.7%) cases exhibited stage I disease, 97 cases (62.2%) exhibited stage II disease and 36 cases (23.1%) presented with stage III. The median follow-up time was 61 months (range, 7–94 months). No patients were lost to follow-up. A total of 132 patients (84.6%) received adjuvant chemotherapy, including anthracycline-based compounds (111 cases in total; 98 cases received anthracycline drugs + cyclophosphamide + fluorouracil, and 13 cases received anthracyclines + paclitaxel) or a cyclophosphamide + methotrexate + fluorouracil-based regimen for 4–8 cycles (21 cases). Additionally, 33 cases (21.2%) received adjuvant radiotherapy (60Co or linear accelerator) at a dose 50 Gy, or 60 Gy for breast-conserving surgery. A total of 46 cases (29.5%) exhibited recurrent metastasis. Of these, 19 cases exhibited metastasis to the lung, 16 cases exhibited liver metastasis, 9 cases exhibited bone metastasis and two cases exhibited brain metastasis. A total of 43 mortalities occurred, including five mortalities due to non-cancer-associated causes, such as cardiovascular disease (Table I).

### Association between MDR protein expression and clinicopathological variables, including HER-2, ER, PR and p53 status

**Figure 1:**
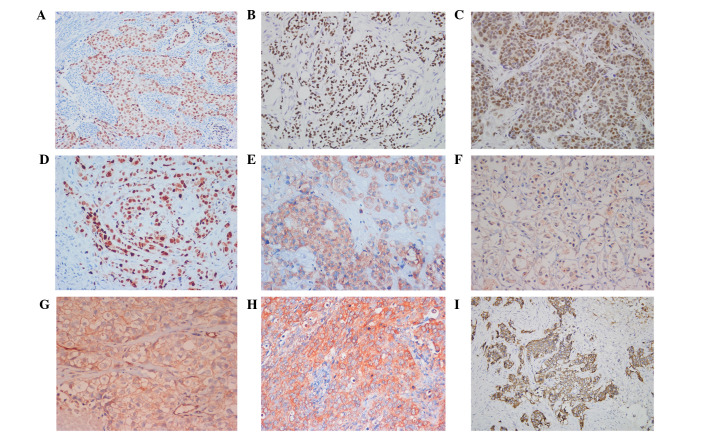
Positive immunohistochemical staining of (A) ER, (B) PR, (C) p53, (D) Topo2α, (E) GST-π, (F) TS, (G) P-gp and (H) MRP, and (I) HER-2 overexpression in invasive ductal carcinoma of breast (streptomycin avidin-peroxidase, magnification, ×200). ER, estrogen receptor; PR, progesterone receptor; Topo2α, topoisomerase 2α; GST-π, glutathione-*S*-transferase; TS, thymidylate synthase; P-gp, p-glycoprotein; MRP, multidrug resistance-related protein; HER-2, human epidermal growth factor receptor 2.

The expression of MRP, P-gp, Topo2α, TS and GST-π was detected in 20.5% (32/156), 25.0% (39/156), 84.0% (131/156), 41.7% (65/156) and 41.0% (64/156) of cases examined, respectively. Representative staining for each of the aforementioned proteins are shown in Fig. 1. Pearson χ^2^ analysis revealed that MRP and Topo2α protein expression did not correlate with patient age, tumor size, axillary lymph node metastasis, histological grade, HER-2 overexpression or expression status of ER, PR and p53. P-gp expression was significantly higher in grade III tumors compared with grade I (χ^2^=16.060; P<0.001) and grade II (χ^2^=13.563; P<0.001) tumors. No significant difference in GST-π staining was identified between grade I and II tumors (χ^2^=2.492; P=0.114), however, there was a significant difference between tumors of grades I and III (χ^2^=16.001; P<0.001) and II and III (χ^2^=34.998; P<0.001). GST-π expression was highest in grade III IDC breast tissue. GST-π expression was also increased in ER-negative tumors (65.3%; χ^2^=17.407; P<0.001) with a Spearman’s correlation coefficient of −0.437 (P<0.001). TS expression (74.6%; 44/59 cases) was increased in breast cancer cases with axillary lymph node metastasis (χ^2^=42.281; P<0.001) (Table I).

### Association between the expression of MRP, TS, Topo2α, P-gp and GST-π proteins

Pearson’s χ^2^ test was performed to examine the associations between the five MRPs. No significant correlation was identified between MRP protein expression and the expression of TS (χ^2^=3.523; P=0.061), Topo2α (χ^2^=2.409; P=0.121), P-gp (χ^2^=3.355; P=0.067) or GST-π (χ^2^=2.769; P=0.096). Furthermore, no significant association was identified between TS expression and the expression of Topo2α (χ^2^=1.308; P=0.253), P-gp (χ^2^=1.064; P=0.302) or GST-π (χ^2^=2.047; P=0.153). Similarly, no significant correlation was identified between Topo2α and GST-π (χ^2^=0.599; P=0.439) or P-gp (χ^2^=0.397; P=0.529). However, a significant positive correlation was identified between P-gp and GST-π (χ^2^=20.348; P<0.001) with a Spearman’s correlation coefficient of 0.319 (P<0.001).

### TS and GST-π expression are associated with poor overall survival

**Figure 2:**
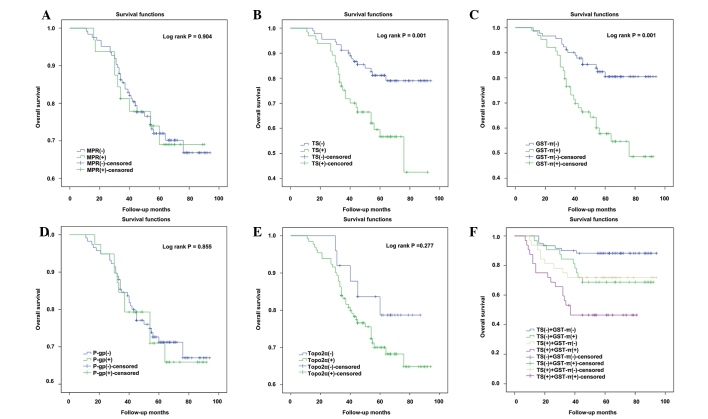
Analysis of the correlations between the five multidrug resistance proteins (A) MRP (B) TS (C) GST-π (D) P-gp (E) Topo2α and (F) combined expression of TS and GST-π and overall survival. Patients with positive TS and GST-π expression exhibited a significantly shorter overall survival time than patients with negative expression for the two proteins. MRP, multidrug resistance-related protein; TS, thymidylate synthase; GST-π, glutathione-*S*-transferase; P-gp, p-glycoprotein; Topo2α, topoisomerse2α.

Positive staining of MRP, P-gp and Topo2α were not found to significantly correlate with changes in overall survival (Fig. 2)*.* Kaplan-Meier survival analyses revealed that patients with tumor specimens that stained positive for TS expression exhibited significantly poorer overall survival rates than patients with TS-negative tumors (P=0.001; Fig. 2B). Similarly, patients with GST-π-positive tumors had a poorer overall survival rate compared with patients with GST-π-negative breast tumors (P=0.001; Fig. 2C). TS and GST-π were then examined to determine whether they represent independent prognostic factors in the disease. As shown in Table II, Cox univariate analysis revealed that positive staining for TS or GST-π was associated with a significantly increased risk of mortality in breast carcinoma patients (TS, P=0.002; GST-π, P=0.001). These factors were also positively associated with histological grade (P<0.001). Cox multivariate analysis indicated that TS and GST-π were independent prognostic factors (P=0.018 and P=0.001, respectively) and that tumor grade was a predictor of a poor survival outcome (P<0.001).

## Discussion

MDR is a common mechanism by which tumor cells become resistant to numerous chemotherapeutic agents. While this MDR response is multi-faceted, it typically involves the upregulation of several key proteins that promote the efflux of drugs out of tumor cells, decreasing their biological efficacy. In the present study, the expression of several key MDR-associated proteins, including MRP, P-gp, Topo2α, TS and GST-π, was investigated in IDC of the breast. The study analyzed the expression of these factors together with several clinicopathological variables and overall survival. We hypothesized that the expression of these factors may be useful as biomarkers of the disease and may explain the occurrence of chemotherapy resistance.

The expression of one of the proteins examined, P-gp, was significantly higher in grade III tumors compared with grade I and II tumors. Previous studies have demonstrated that this protein promotes the efflux of a number of anticancer drugs, including anthracyclines, vinca alkaloids, taxanes, epipodophyllotoxins and doxorubicin, out of tumor cells (7,8). P-gp is also involved in the secretion of anticancer agents into bile, urine and the intestinal lumen, which markedly affects the pharmacokinetic properties and bioavailability of therapeutically administered compounds (9). Linn *et al* (10) evaluated P-gp expression in 92 primary and 12 metastatic breast cancers and found that P-gp expression was associated with a poor prognosis. In another study, metastatic breast cancer patients negative for P-gp expression (P=0.06) exhibited a longer progression-free survival time following docetaxel treatment compared with patients exhibiting P-gp-positive tumors (11). In the present study, P-gp and GST-π expression were found to be positively correlated (r=0.319; P<0.0001). Additionally, the expression of the two proteins was elevated in grade III tumors. Consistent with the findings of the present study, Cui *et al* (12) identified a positive correlation (r=0.429; P<0.01) between P-gp and GST-π in 76 breast cancer patients prior to treatment. Furthermore, another study investigated the correlation between the expression of P-gp, GST and metallothioneins (MTs) and the response to various chemotherapy regimens in triple--negative (ER-, PR- and HER2-negative) breast cancer patients (13). The chemotherapy-treated groups demonstrated improved three-year relapse-free survival rates (P<0.05), which were associated with the expression of P-gp, GST and MT. These results, in addition to the results of the present study, indicate a function for these MRPs in the outcome of breast cancer and its response to chemotherapy.

As aforementioned, GST-π expression was highest in grade III IDC breast tumors and also increased in ER-negative disease. Notably, patients with tumors staining positive for GST-π were also found to exhibit poorer overall survival rates than patients with GST-π-negative tumors. Thus, the results of this study indicated that GST-π is associated with a worse prognosis and may potentially drive disease progression. These findings are supported by previous studies. For example, resistance against drugs and environmental insults is conferred by the glutathione metabolic pathway (4). The GSTs are a family of enzymes involved in the metabolism of a broad range of xenobiotics, which have been shown to inactivate platinum drugs, doxorubicin, cyclophosphamide and etoposide (14,15). In a previous study, the expression of GST-π was investigated in 21 primary untreated human breast tumors (16) and in agreement with the findings of the present study, the mean expression of GST-π in ER-negative tumors was found to be 5-fold greater than the mean expression in ER-positive tumors. These findings were also consistent with another study examining 189 breast cancer cases (17). Overall, patients with ER-negative tumors may exhibit increased resistance to chemotherapeutic regimens due to increased GST-π levels. The results of the present study indicate an association between ER and GST-π, which requires additional investigation in the future.

Topo2 is an essential nuclear DNA-binding enzyme that controls and modifies the topological states of DNA by combining nuclease, helicase and ligase activities (18). Topo2α is a specific isoform that is located on chromosome 17q21 in close proximity to HER-2. The exact association between Topo2α and HER-2 remains unclear. While certain studies have found no association between Topo2α and HER2 overexpression (19–21), other studies have reported that the increased expression of Topo2α is associated with HER-2 amplification or overexpression (22,23). To better define the clinical relevance of Topo2α expression, larger prospective studies are required. In this study, positive staining for Topo2α was identified in 84.0% (131/156) of the cases examined, indicating that Topo2α expression may be significant in breast cancer. Notably, Mukherjee *et al* (24) revealed that the expression of Topo2α prior to the administration of chemotherapy significantly correlated with the pathological complete response to neoadjuvant anthracycline treatment. Another study reported that ER is an independent predictive factor for pathological response to three different pre-operative chemotherapy regimens in primary breast tumors (25), however, the expression of PR, Topo2, P-gp, MRP and GST-π were not predictive of the pathological response to the three treatment regimens.

TS is a folate-dependent enzyme involved in pyrimidine synthesis that is crucial for cellular proliferation and growth (26). TS also catalyzes the methylation of deoxyuridine monophosphate to deoxythymidine monophosphate, an essential precursor of DNA biosynthesis (27). In the present study, TS expression was found to be elevated in cases of invasive breast carcinoma with lymph node metastasis. Thus, these breast cancers are more aggressive and exhibit a poorer overall prognosis. Furthermore, a similar association between TS levels and prognosis has been reported in other tumor types, including colorectal, rectal and gastric cancers (28–30). TS overexpression is a biomarker of 5-fluorouracil (5-FU) resistance in human cancer cells (31). However, 5-FU combined with low-dose trichostatin A (50 nmol/l) has been shown to restore 5-FU-mediated cytotoxicity in 5-FU-resistant cancer cells in combination with the downregulation of TS protein expression (31). In another study of advanced-stage breast cancer patients, lower TS levels were associated with an improved response to the chemotherapy drug pemetrexed. Additionally, in a number of patients, continuous administration of pemetrexed has been found to decrease TS levels (32). Brandi *et al* (33) revealed that patients with low levels of TS and high levels of p53 responded better to docetaxel. Overall, these results indicate that low levels of TS may be associated with an improved breast cancer prognosis and response to chemotherapy administration.

In conclusion, the assessment of MDR protein expression in breast cancer may be a useful predictor of prognosis and the response to chemotherapy. Ultimately, this information may aid clinicians in the design of unique treatment regimens for each individual patient.

## Figures and Tables

**Table I:** Correlation between MRP, P-gp, Topo-2α, TS and GST-π and the clinicopathological variables and status of ER, PR, HER2 and p53.

		MRP			P-gp			Topo-2α			TS			GST-π		
						
Variable	n	+/++/+++, n (%)	χ^2^	P-value	+/++/+++, n (%)	χ^2^	P-value	+/++/+++, n (%)	χ^2^	P-value	+/++/+++, n (%)	χ^2^	P-value	+/++/+++, n (%)	χ^2^	P-value
Age, years			0.177	0.674		0.556	0.456		1.626	0.202		1.442	0.230		0.032	0.859
<50	68	15 (22.1)			19 (27.9)			60 (88.2)			32 (47.1)			27 (39.7)		
≥50	88	17 (19.3)			20 (22.7)			71 (80.7)			33 (37.5)			37 (42.0)		
Tumor size, cm			1.546	0.214		1.074	0.300		1.897	0.168		1.979	0.160		0.891	0.345
≤2.0	63	16 (25.4)			13 (20.6)			56 (88.9)			22 (34.9)			23 (36.5)		
>2.0	93	16 (17.2)			26 (28.0)			75 (80.7)			43 (46.2)			41 (44.1)		
Lymph nodes			1.403	0.236		3.280	0.070		0.060	0.806		42.281	0.000^a^		0.071	0.790
Negative	97	17 (17.5)			29 (29.9)			82 (84.5)			21 (21.6)			39 (40.2)		
Positive	59	15 (25.4)			9 (15.3)			49 (83.1)			44 (74.6)			25 (42.4)		
Clinical Stages			0.693	0.707		0.789	0.674		2.548	0.280		1.450	0.484		0.164	0.921
I	23	5 (21.7)			7 (30.4)			18 (78.3)			8 (34.8)			9 (39.1)		
II	97	18 (18.6)			22 (22.7)			85 (87.6)			44 (45.4)			41 (42.3)		
III	36	9 (25.0)			10 (27.8)			28 (77.8)			13 (36.1)			14 (38.9)		
Histological grade			0.080	0.961		20.226	0.000^a^		4.460	0.108		3.855	0.146		35.032	<0.001^a^
I	37	7 (18.9)			4 (10.8)			27 (73.0)			11 (29.7)			15 (40.5)		
II	91	19 (20.9)			19 (20.9)			79 (86.8)			39 (42.9)			24 (26.4)		
III	28	6 (21.4)			16 (57.1)			25 (89.3)			15 (53.6)			25 (89.3)		
ER status			0.685	0.164		0.010	0.921		1.799	0.180		2.572	0.109		17.407	<0.001^a^
+/++/+++	107	21 (19.6)			27 (25.2)			87 (81.3)			40 (37.4)			32 (29.9)		
−	49	11 (22.4)			12 (24.5)			44 (89.8)			25 (51.0)			32 (65.3)		
PR status			0.288	0.592		3.174	0.075		2.288	0.130		2.623	0.105		3.101	0.078
+/++/+++	91	20 (22.0)			18 (19.8)			73 (80.2)			33 (36.3)			32 (35.2)		
−	65	12 (18.5)			21 (32.3)			58 (89.2)			32 (49.2)			32 (49.2)		
HER-2 status			0.003	0.959		2.261	0.133		0.109	0.741		0.012	0.912		3.026	0.082
2+/3+	64	13 (20.3)			20 (31.3)			53 (82.8)			27 (42.2)			21 (32.8)		
0/1+	92	19 (20.7)			19 (20.7)			78 (84.8)			38 (41.3)			43 (46.7)		
p53 status			1.887	0.170		0.103	0.749		0.143	0.705		0.712	0.399		1.272	0.259
+/++/+++	117	27 (23.1)			30 (25.6)			99 (84.6)			51 (43.6)			45 (38.5)		
−	39	5 (12.8)			9 (23.1)			32 (82.1)			14 (35.9)			19 (48.7)		

a P<0.05.

MRP, multidrug resistance-related proteins; P-gp, p-glycoprotein; Topo-2α, topoisomerase 2α; TS, thymidylate synthase; GST-π; glutathione-*S*-transferase π; ER, estrogen receptor; PR, progesterone receptor; HER-2, human epidermal growth factor receptor 2.

**Table II:** Cox univariate and multivariate analysis of five-year overall survival on MDR proteins and clinicopathological variables.

MDR proteins and clinicopathological variables	Univariate		Multivariate	
	
	HR (95% CI)	P-value	HR (95% CI)	P-value
MRP	1.023 (0.739–1.865)	0.905	1.281 (0.806–2.036)	0.295
TS	1.634 (0.988–2.161)	0.002^a^	1.481 (1.070–2.048)	0.018^a^
Topo2α	1.291 (0.809–2.059)	0.284	1.557 (0.949–2.555)	0.080
P-gp	1.032 (0.845–1.096)	0.855	0.753 (0.506–1.120)	0.161
GST-π	1.683 (0.917–2.325)	0.001^a^	1.853 (1.284–2.674)	0.001^a^
Age	0.976 (0.872–1.085)	0.115	1.016 (0.966–1.069)	0.538
Menstrual	1.789 (0.645–7.016)	0.060	1.862 (0.551–6.295)	0.317
Tumor size	1.002 (0.502–1.592)	0.995	0.959 (0.482–1.908)	0.905
Lymph node	1.660 (0.635–3.194)	0.098	1.528 (0.719–3.243)	0.271
Histological grade	3.471 (1.122–4.125)	<0.001^a^	3.089 (1.819–5.243)	<0.001^a^

a P<0.05 was considered to indicate a statistically significant difference.

MDR, multidrug resistance; TS, thymidylate synthase; P-gp, p-glycoprotein; Topo2α, topoisomerase 2α; GST-π, glutathione-*S*-transferase π; CI, confidence interval.
